# Assessment of the Amount of Calcium Ions Released after the use of Different Chelating Agents and Agitation Protocols

**DOI:** 10.2174/1874210601711010133

**Published:** 2017-03-22

**Authors:** Fábio Luis Miranda Pedro, Laura Maria Amorim Santana Costa, Gilberto Siebert Filho, Orlando Aguirre Guedes, Thiago Machado Pereira, Alvaro Henrique Borges

**Affiliations:** Department of Oral Sciences, University of Cuiabá, Cuiabá-MT, Brazil

**Keywords:** Chelating agents, Chitosan, EDTA, Endodontics, Irrigants, Ultrasonic

## Abstract

**Background:**

The main goal of endodontic treatment is to achieve cleaning and shaping prior to the filling process.

**Objective:**

This study aimed to evaluate, using atomic absorption spectrometry, the release of Calcium ions after the use of different chelating agents and protocols of agitation.

**Method:**

Ninety human canine teeth were randomly assigned to one of nine groups (n=10), as follows: 1) 0.2% Chitosan and manual agitation; 2) 0.2% Chitosan and sonic agitation; 3) 0.2% Chitosan and ultrasonic agitation; 4) 17% EDTA and manual agitation; 5) 17% EDTA and sonic agitation; 6) 17% EDTA and ultrasonic agitation; 7) distilled water and manual agitation; 8) distilled water and sonic agitation; 9) distilled water and ultrasonic agitation. Following instrumentation, all chelating substances remained inside the root canal for 3 min. Then the fluid was collected for the identification and quantification of Calcium ions. The amount of Calcium ions released in each group was compared using analysis of variance (ANOVA) and the Kolmogorov-Smirnov and Levene tests followed by Tukey’s post-hoc test. Significance was set at 5%.

**Results:**

The groups in which 0.2% Chitosan was used showed the highest concentration of Calcium ions (p<0.05). Concerning the agitation method, ultrasonic agitation showed the greatest values, followed by sonic and manual agitation (all comparisons, p<0.05).

**Conclusion:**

The present findings suggest that, among the combinations here tested, Chitosan associated with ultrasonic agitation yielded the greatest release of Calcium ions.

## INTRODUCTION

The main goal of root canal treatment is to achieve optimal cleaning and shaping conditions prior to the filling process [1]. In this scenario, biomechanical preparation is paramount when endodontic instruments are used in association with auxiliary chemical substances [2, 3]. Regardless of the instrument and technique employed, the action of endodontic instruments against dentinal walls inevitably promotes the formation of a smear layer of granular appearance, containing both inorganic and organic materials [2, 4, 5]. Smear layer removal is strictly related to treatment success, and has been shown to be directly correlated with the irrigation process, and consequently with neutralization of the root canal microbiota [4, 6-8].

Sodium hypochlorite (NaOCl) is a widely used endodontic irrigant, especially due to its antimicrobial properties and the ability to dissolve organic materials [7]. However, it does not act on the inorganic contents of the smear layer, and therefore requires the associated use of a chelating agent [7, 8]. Some chelating agents have the ability to bind to metallic ions of a particular molecular complex, among those agents, the use of ethylenediaminetetraacetic acid (EDTA) is a consensus [2, 9, 10]. The reaction with Calcium ions in dentin results in Calcium chelation, with decalcification of the dentin structure [10]. However, the combined use of irrigating and chelating solutions promotes smear layer removal and microhardness reduction and can enhance dentin erosion [11].

Biocompatible solutions can minimize the aggression to dental structures. In this scenario, options such as Citric acid, Lactic acid, and apple vinegar have emerged as potential substitutes for traditional chelators/EDTA [9, 12, 13]. Chitosan, for instance, is a natural polysaccharide that has been tested as an alternative material in dentistry, due to its biocompatibility, biodegradability and bioadhesion in the human body [14]. This substance is produced through deacetylation of chitin, obtained from shrimp and crab shells [14,15]. In endodontics, it has been used as an intracanal medication in association with Calcium Hydroxide paste and as a chelating agent to help reduce root dentin microhardness [3, 4, 16].

Dentin permeability affects the action of intracanal medications and especially the filling of the root canal system [10]. There is controversy in the literature regarding the application time of irrigating and chelating substances [17]. It is known that these agents are used in root canals in different ways, with or without agitation (hand files, motor-driven systems, lentullo files, sonic and ultrasonic systems), as supplementary agents to improve results [18, 19]. Thus, the aim of this study was to assess, using atomic absorption spectrometry, the amount of Calcium ions released after the use of 0.2% Chitosan and 17% EDTA associated with different protocols of agitation.

## MATERIALS AND METHODOLOGY

The study protocol was approved by the Research Ethics Committee of University of Cuiabá, Cuiabá, Brazil (CAAE 44419815000005165). Ninety extracted human maxillary canines, single-rooted, with fully formed apices and straight root canals (r<5) [20], were used. The teeth exhibited no defects, no calcifications, no internal or external root resorption, no prosthetic crowns or dental posts, no previous root canal treatment and no aberrant canal morphology. All teeth had a single canal and a single apical foramen as evidenced by buccal and proximal radiographic examinations. Root apices were inspected under a stereoscopic microscope (Expert DN; Müller Optronic, Erfurt, Germany), and root canals were explored with a #.08 K-file (Dentsply Maillefer, Ballaigues, Switzerland). To improve standardization, only teeth measuring between 20 and 22 mm confirmed using a millimeter ruler (Dentsply Maillefer, Ballaigues, Switzerland) were included in the study. The diameter of the foramina of all teeth was standardized using a #.15 K-file (Dentsply Maillefer, Ballaigues, Switzerland). Specimens were disinfected using 2.5% NaOCl and stored in 0.1% thymol solution at 4°C until use.

Standard access cavities were made using round diamond burs (#.1011 and #.1012; KG Sorensen, Barueri, SP, Brazil) coupled to a high speed handpiece with air/water spray cooling. The apical patency of all root canals was confirmed using a #.10 K-file (Dentsply Maillefer, Ballaigues, Switzerland), and canals with a patency greater than ISO 15 were discarded. Working length was determined using a #.15 K-file (Dentsply Maillefer, Ballaigues, Switzerland), which was introduced into the root canal until its tip became visible at the apical foramen using an operating microscope (OPMI Pico; Carl Zeiss, Oberkochen, Germany). Working length was set to 1 mm short of that length.

Specimens were randomly assigned to two broad experimental groups according to the chelating solution employed (0.2% Chitosan or 17% EDTA); there was also a control group in which distilled water was used. Then, the groups were randomly assigned to three subgroups, according to the protocol of agitation (manual, sonic, and ultrasonic agitation). The final groups were as follows: 1) 0.2% Chitosan and manual agitation; 2) 0.2% Chitosan and sonic agitation; 3) 0.2% Chitosan and ultrasonic agitation; 4) 17% EDTA and manual agitation; 5) 17% EDTA and sonic agitation; 6) 17% EDTA and ultrasonic agitation; 7) distilled water and manual agitation; 8) distilled water and sonic agitation; and 9) distilled water and ultrasonic agitation.

Specimens were instrumented using a #.40.08 WaveOne Large-file (Dentsply-Maillefer, Ballaigues, Switzerland) activated by an X-Smart Plus motor (Dentsply-Maillefer, Ballaigues, Switzerland) set according to manufacturer instructions. For cervical enlargement, a #.02 LA Axxess bur (SybronEndo, Orange, CA, USA) was used, driven by Intramatic 2068 (Kavo, Joinvile, Santa Catarina, Brazil) and Intramatic 181DBN micromotors (Kavo, Joinvile, Santa Catarina, Brazil) operating at 5000 rpm. The LA Axxess bur was used until the operator felt resistance to penetrate. Final preflaring depths ranged from 12 to 14 mm. Four milliliters of 2.5% NaOCl was used as irrigant after each instrument. A NaviTip 31ga (Ultradent, South Jordan, UT, USA) irrigation needle was used which was 1 mm short of the working length. Final irrigation was performed with 3 ml of bi-distilled water. Patency was assessed using a #.10 K-file. Each instrument was used to prepare one root canal and then discarded. All root canals were prepared by a single experienced endodontist.

Subsequently, canals were irrigated and filled with 5 ml of one of the solutions investigated: 17% EDTA (Biodinâmica, Ibiporã, PR, Brazil), 0.2% Chitosan (Naturallis, Várzea Grande, MT, Brazil), or distilled water, as a final irrigant. Then, the solutions were agitated for 3 min according to the protocol of agitation assigned (manual, sonic, or ultrasonic). In the groups subjected to sonic agitation, the EndoActivator^TM^ device (Dentsply, Tulsa Dental Specialities, USA) with a #.35/04 tip was used. The device was introduced up to 2 mm short of the working length. In the ultrasonic groups, the chelating solution was activated using a #.15 file (Satelec, Acteon, France) driven by an ultrasonic device (Piezo-Electric MTS; Multi Task Cart, Obtura Spartan, USA) at 4/10-scale power in accordance with manufacturer instructions. Finally, in the manual groups, the chelating solutions were agitated manually using a #.40 K-file (Dentsply-Maillefer, Ballaigues, Switzerland). In all groups, following activation, root canals were washed with saline solution and dried with paper points.

Extruded debris and irrigant solutions were collected in a container fitted with a rubber stopper and individually prepared to support each tooth with its apex suspended within the container, according to the method described by Myers and Montgomery [21]. Each specimen was attached to the rubber stopper using cyanoacrylate-based adhesive (Super Bonder; Loctite of Brazil, Henkel Ltda., São Paulo, SP, Brazil), and 1/2 Q1 sealing tape (Tigre AS, Joinville, SC, Brazil), which was used to protect the specimen rubber stopper interface. The container was vented with a 25-G needle (Injex Indústria Cirúrgica Ltda., Ourinhos, SP, Brazil) inserted through the rubber stopper to equalize pressures. Once instrumentation was completed, teeth were separated from the container. Debris adhered to the surface of the root were collected by rinsing the specimens with 1 ml of Bi-distilled water. Containers were then placed in an incubator at 70°C for 5 days for moisture evaporation before weighing the debris.

### Concentration of Calcium Ions (Atomic Absorption Spectrometry)

The contents extruded from the instrumented tooth were transferred to a 100-ml Teflon tube. A mixture of 7 ml of Nitric acid (65% by volume) and 21 ml of Hydrochloric acid (37% by volume) was added to the Teflon tube and kept aside for 2 hours. After that, the tube was loosely capped and transferred to a hood provided with exhaust ventilation. The tube was placed on a heating block, heated to 80°C and kept heated for 150 min to allow the temperature to stabilize. After the reaction, the tube was cooled to room temperature, and the mixture was filtered using grade 40 paper filter (Whatman, GE, Healthcare Life Sciences, Pittsburgh, PA, USA); distilled water was added to the resulting filtrate until a volume of 50 ml was reached. A blank test was performed in parallel, using the same procedure and the same amounts of all reagents, but omitting the test specimen.

For the analysis of Calcium ion concentration, a specific pattern was determined from universal standards (Merck, Darmstadt, Germany). Micropipettes (Boeco, Germany) with adjustable volumes of 5-50 µL, 50-200 µL, and 100-1000 µL were used for the preparation of standard solutions and specimens. The standard was prepared by adding appropriate amounts of aqueous analyte stock solutions plus 1 g of material to 10-ml volumetric flasks, which were then completed with Bi-distilled water. Bi-distilled water was used for adjustment, and the washing procedure was performed in a 250-ml separatory funnel to which 50 ml of Bi-distilled water and 100 ml of a solution of 1.0% (v/v) Nitric acid were added; the resulting mixture was heated to 70°C. Then the separatory funnel was shaken vigorously for 5 min, and the extraction procedure was repeated until no analytes were detected in the specimen using the flame atomic absorption spectrometer (Varian Ind. Com. Ltda., São Paulo, SP, Brazil). Analytical curves were prepared in the concentration range of 0.0-0.8 mg L^−1^, using Bi-distilled water as medium. The reading parameter for Calcium was determined considering the electric current, wavelength, and slit aperture of the device.

The amounts of Calcium ions released in the different groups were compared using analysis of variance (ANOVA) and the Kolmogorov-Smirnov and Levene tests followed by Tukey’s post-hoc test. Significance was set at p<0.05. Statistical analyses were performed using the Statistical Package for the Social Sciences (SPSS) version 18.0 for Windows (SPSS Inc., Chicago, IL, USA).

## RESULTS

Considering the chelating agents tested, 0.2% Chitosan showed the highest amount of Calcium ions released (168.38 ± 6.52), 17% EDTA showed intermediate results (58.59 ± 26.20), and distilled water showed the lowest values (2.52 ± 0.86). Differences were significant across all three groups (*P*<0.05).

Table **1** shows the results obtained with the different protocols of agitation combined with different chelating agents. Considering the different protocols of agitation tested, ultrasonic agitation yielded the highest results, followed by sonic agitation and manual agitation, respectively (all comparisons, *P*<0.05). Within each agitation group, association with 0.2% Chitosan always showed significantly higher values (*P*<0.05).

## DISCUSSION

Several advances in dentistry originate from scientific developments in the biomaterials sector, particularly with the release of materials for use in dental practice [22]. In the present study, the decalcification properties of two chelators were evaluated when associated with different protocols of agitation. Chelating agents play an important role in the process of removing smear layer, as they increase dentin permeability [23, 24]. We assessed the effectiveness of two chelating substances (0.2% Chitosan and 17% EDTA) based on the amount of Calcium ions released, using atomic absorption spectrometry. This tool allowed to quantify the concentration of Calcium ions present in the substance after irrigation, as previously used by other authors [12].

The Calcium ions present in the dentin are released when in contact with chelating agents [2, 23]. Smear layer removal by 17% EDTA combined with 5% NaOCl [2] is known to reduce root dentin microhardness, thus facilitating instrumentation [3]. However, EDTA has an erosive effect on dentin and attacks periapical tissues; in addition, it is considered a pollutant [24].

In the present study, 0.2% Chitosan was associated with the highest amounts of Calcium ions released, followed by 17% EDTA and distilled water, respectively (all *P*<0.05). EDTA and Chitosan are known to act on root dentin demineralization, which is in line with the results of the present study [25]. Concerning the application time of chelating agents, the use of solutions for more than 5 min has been shown not to have an effect on their ability to remove Calcium [26]. Solutions applied for 3 min, in turn, have proved effective in removing smear layer and smear plug, with minimal erosive effect [24].

Chitosan presents some other advantages over EDTA: it is biocompatible, biodegradable, bioadhesive, and atoxic [27]. It could be used as a final irrigant, as it may act as both a chelating agent and an antibiofilm agent [28]. In addition, 0.2% Chitosan is efficient in removing smear layer from the middle and apical thirds of root canals (demineralization capacity) [25]. Another major advantage of Chitosan is its ability to control the release of Calcium ions from Calcium Hydroxide when used as a vehicle, for up to 30 days [16]. Finally, it has the capacity to remineralize the dentin, allowing for the investigation of new types of therapies [29].

Regarding the other variable analyzed in this study, namely, mechanical agitation of chelating substances, ultrasonic agitation showed the highest amount of Calcium ions released in the spectrometric analysis. This result is closely related to the intensity of the movement produced inside the root canal, as suggested by some previous studies [3, 30, 31]. The mechanisms of ultrasonic agitation are related to acoustic microstreaming and Hydrodynamic cavitation, which result in formation and implosion of vapor bubbles; sonic activation, in turn, cannot promote cavitation as a result of the low oscillation speed [32, 33].

Lui, *et al*. [34] described the use of ultrasonic irrigation following instrumentation/during the final irrigation and found that ultrasonics improved smear layer removal, which corroborates the findings of this study. The protocols employed by the two studies are different, especially with regard to application time: whereas Lui *et al.* used 1 min of ultrasonic irrigation with 17% EDTA and obtained clean, smear-free dentinal walls [34], in this study we used 0.2% Chitosan and 17% EDTA for 3 min. Nevertheless, the results obtained were similar. It seems that the ultrasonic technique alone or combined with NaOCl is unable to obtain good results [35]. Conversely, some chelators can have their ability to remove smear layer enhanced by ultrasonic devices [34]. Considering the satisfactory results obtained in this study with 0.2% Chitosan combined with ultrasonic agitation, and the superior characteristics of this biopolymer [2, 3, 12, 24], further studies (especially with a focus on cytotoxicity and genotoxicity) are important to investigate the use of this substance as an irrigating agent.

## CONCLUSION

Considering the experimental conditions of this study, 0.2% Chitosan combined with ultrasonic agitation yielded the highest amounts of Calcium ions released.

## Figures and Tables

**Table 1:** Amount of Calcium Ions released (in mg/L) according to agitation protocol and chelating agent employed.

	**Chelating agent**		
**Agitation protocol**	**Distilled water**	**0.2% Chitosan**	**17% EDTA**
**Manual**	1.52 ± 0.07^Aa^	161.96 ± 2.18^Ab^	25.10 ± 3.15^Ac^
**Sonic**	2.55 ± 0.35^Ba^	166.94 ± 2.19^Bb^	64.95 ± 2.74^Bc^
**Ultrasonic**	3.49 ± 0.13^Ca^	176.24 ± 2.80^Cb^	85.74 ± 3.45^Cc^

Data presented as means ± standard deviation. * Different upper-case letters indicate statistically significant differences (*P*<0.05) within columns. * Different lower-case letters indicate statistically significant differences (*P*<0.05) within rows.
